# Detailed Characterization of Early HIV-1 Replication Dynamics in Primary Human Macrophages

**DOI:** 10.3390/v10110620

**Published:** 2018-11-10

**Authors:** David Alejandro Bejarano, Maria C. Puertas, Kathleen Börner, Javier Martinez-Picado, Barbara Müller, Hans-Georg Kräusslich

**Affiliations:** 1 Department of Infectious Diseases, Virology, University of Heidelberg, 69120 Heidelberg, Germany; david.bejarano@med.uni-heidelberg.de (D.A.B.); kathleen.boerner@bioquant.uni-heidelberg.de (K.B.); barbara.mueller@med.uni-heidelberg.de (B.M.); 2 AIDS Research Institute IrsiCaixa, Institut d’Investigació en Cièncias de la Salut Germans Trias i Pujol, Universitat Autònoma de Barcelona, 08916 Badalona, Spain; mcpuertas@irsicaixa.es (M.C.P.); jmpicado@irsicaixa.es (J.M.-P.); 3 German Center for Infection Research, Partner Site Heidelberg, 69120 Heidelberg, Germany; 4 Faculty of Medicine, University of Vic—Central University of Catalonia (UVic-UCC), 08500 Vic, Spain; 5 Catalan Institution for Research and Advanced Studies (ICREA), 08010 Barcelona, Spain

**Keywords:** human immunodeficiency virus, primary macrophages, reverse-transcription complex, pre-integration complex, replication kinetics, SAMHD1

## Abstract

Macrophages are natural target cells of human immunodeficiency virus type 1 (HIV-1). Viral replication appears to be delayed in these cells compared to lymphocytes; however, little is known about the kinetics of early post-entry events. Time-of-addition experiments using several HIV-1 inhibitors and the detection of reverse transcriptase (RT) products with droplet digital PCR (ddPCR) revealed that early replication was delayed in primary human monocyte-derived macrophages of several donors and peaked late after infection. Direct imaging of reverse-transcription and pre-integration complexes (RTC/PIC) by click-labeling of newly synthesized DNA further confirmed our findings and showed a concomitant shift to the nuclear stage over time. Altering the entry pathway enhanced infectivity but did not affect kinetics of viral replication. The addition of viral protein X (Vpx) enhanced productive infection and accelerated completion of reverse transcription and nuclear entry. We propose that sterile alpha motif (SAM) and histidine/aspartate (HD) domain-containing protein 1 (SAMHD1) activity lowering deoxyribonucleotide triphosphate (dNTP) pools is the principal factor delaying early HIV-1 replication in macrophages.

## 1. Introduction

Human immunodeficiency virus type 1 (HIV-1) primarily infects and replicates in cluster of differentiation 4 positive (CD4^+^) T cells. However, macrophages also constitute a relevant natural target cell of the virus and are a crucial vehicle of viral spread within the infected organism [1]. In contrast to lymphocytes, they are largely refractory to the cytopathic effects of productive infection. Accordingly, macrophages can sustain viral replication over extended periods of time in culture and in vivo [2,3,4,5], and are, thus, considered important viral reservoirs.

Kinetics of individual HIV-1 replication steps in T-cell lines and primary CD4^+^ lymphocytes were previously analyzed in detail (e.g., [6,7,8,9]). A particular focus of interest was on early post-entry events—from cytosolic entry of the viral capsid over reverse transcription of the viral genome to its transport to and integration into the host cell DNA—which establish persistent infection and are key targets of intrinsic cellular restriction factors. A recent report analyzed the timing of the various stages of viral replication at the single-cell level in the MT-4 T-cell line. Reverse transcription of the viral RNA genome was found to be completed within ca. 14 h post infection (p.i.), while integration of the resulting viral complementary DNA (cDNA) into the host cell genome occurred ~19 h p.i., and peak virus production was observed between 30 h and 40 h p.i. [9]. Similarly, early post-entry replication steps in primary lymphocytes were found to occur within the first 24 h of infection, and viral progeny production from these cells was readily detectable at 40 h p.i. [7,10].

In contrast, HIV-1 replication kinetics in primary macrophages are much less characterized. Productive HIV-1 replication in these cells appears to be significantly slower compared to CD4^+^ T lymphocytes. Initial studies of HIV-1 infection of macrophages in vitro using clinical virus isolates and various laboratory strains followed virus production over multiple rounds of replication. These analyses revealed very low levels of p24 capsid (CA) antigen in supernatants two days post infection, and peak levels of virus production from the initial infection were only reached between days five and eight post inoculation [11,12,13]. Furthermore, the efficiency of HIV-1 infection also depends on the pathway of differentiation and type of macrophage polarization [14,15]. Semi-quantitative PCR-based approaches to detect different genome replication intermediates revealed strong-stop cDNA as the first product of HIV-1 reverse transcriptase (RT) at 6 h p.i. in macrophages and T-cell lines alike. However, peak levels of RT products were reached only after 48 h in macrophages, and no evidence for a second round of infection was observed in these cells within the observation time [16,17]. More recent studies reported the appearance of post-integration stages at late times post infection in macrophages (96 h p.i. [18]), also pointing to delayed viral replication. Taken together, the work from many labs indicates slow and inefficient HIV-1 replication in primary macrophages; however, a consistent analysis of individual replication steps throughout several days of infection, as performed in the case of T cells, is missing.

The comparatively low level of deoxyribonucleotide triphosphates (dNTPs) in the cytosol of post-mitotic macrophages was identified as one important reason for the inefficient HIV-1 replication in these cells [19,20,21]. Low dNTP levels are caused, at least in part, by the activity of the cellular phosphohydrolase, sterile alpha motif (SAM) and histidine/aspartate (HD) domain-containing protein 1 (SAMHD1), which is highly expressed in cells of the myeloid lineage [22,23,24,25]. SAMHD1 is counteracted by the viral protein X (Vpx), which is present in HIV-2 and simian immunodeficiency virus (SIV), but not in HIV-1 [26,27]. Accordingly, HIV-2 and SIV replicate more efficiently in primary human macrophages than HIV-1 [28], and HIV-1 replication can be experimentally enhanced by SAMHD1 depletion through knock-down or Vpx expression [25,29,30].

HIV-1 infection of macrophages is frequently scored at 24–48 h, but the respective studies usually employ strategies to enhance infection. Pseudotyping of viral particles with the glycoprotein of vesicular stomatitis virus (VSV-G) directs HIV-1 entry toward clathrin-mediated endocytosis, and significantly enhances the efficiency of macrophage infection [31]. Additionally, or alternatively, intracellular dNTP levels are often manipulated by supplementing deoxyribonucleoside precursors or via Vpx-mediated depletion of SAMHD1. However, the effect of these experimental modifications on the dynamics of individual replication events is largely neglected. Despite the extensive characterization of the effects of Vpx on macrophage infection/transduction and its interaction with SAMHD1 (e.g., [23,25,28,29,32]), the alteration of HIV-1 early post-entry kinetics upon SAMHD1 degradation is yet to be analyzed.

Here, we present a systematic analysis of the kinetics of the early HIV-1 replication steps in primary human monocyte-derived macrophages (MDM) from multiple donors over several days post infection. We also addressed the effects of VSV-G pseudotyping or increasing dNTP availability on the timing of these steps. For this, we complemented inhibitor time-of-addition experiments with sensitive digital droplet PCR (ddPCR) quantitation of different RT products and with imaging of intracellular productive subviral complexes. Early HIV-1 replication peaked at 48–60 h after infection. Time and pathway of entry did not affect the time course of early replication, while treatment with Vpx not only resulted in enhanced reverse transcription, but also accelerated early viral replication. These results indicate that nucleoside triphosphate availability or SAMHD1 activity are the main limiting factors for the early HIV-1 replication steps in macrophages.

## 2. Materials and Methods

### 2.1. Cell Culture

Human embryonic kidney 293T cells (HEK293T) and TZM-bl indicator cells [33,34] were cultured at 37 °C and 5% CO_2_ in Dulbecco’s modified Eagle medium (DMEM; Life Technologies, Carlsbad, CA, USA), supplemented with 10% fetal calf serum (FCS; Biochrom GmbH, Berlin, Germany), 100 U/mL penicillin, and 100 µg/mL streptomycin. For isolation of monocyte-derived macrophages (MDMs), human peripheral blood mononuclear cells (PBMC) were isolated from buffy coats by Ficoll density gradient centrifugation using SepMate tubes (StemCell Technologies, Vancouver, BC, Canada). Buffy coats were obtained from healthy anonymous blood donors at the Heidelberg University Hospital Blood Bank according to the regulations of the local ethics committee. PBMC were seeded in Roswell Park Memorial Institute medium 1640 (RPMI; Life Technologies), supplemented with 10% heat-inactivated FCS and antibiotics, and incubated for 2 h at 37 °C. Cells were washed three times to remove non-adherent cells, and adhered monocytes were subsequently cultured and differentiated into MDMs in RPMI 1640 supplemented with 10% heat-inactivated FCS, antibiotics, and 5% human AB serum (Sigma-Aldrich, St. Louis, MO, USA) for 7–10 days; medium was replaced every three days. In polarization experiments, CD14^+^ monocytes were isolated from PBMC by negative selection using magnetic beads (Monocyte Isolation Kit II; Miltenyi Biotech, Bergisch Gladbach, Germany), seeded in RPMI 1640 supplemented with 10% heat-inactivated FCS and antibiotics, and subsequently differentiated and polarized into M1 or M2a macrophages by adding to the medium 50 ng/mL granulocyte-macrophage colony-stimulating factor (GM-CSF; PeproTech, Hamburg, Germany) and 20 ng/mL interferon gamma (IFNγ; R&D systems, Minneapolis, MN, USA), or 50 ng/mL M-CSF (PeproTech) and 20 ng/mL interleukin 4 (IL-4; R&D systems), respectively.

### 2.2. Plasmids

The proviral HIV-1 plasmid pNL4-3 was described elsewhere [35]. The derivative pNL_4-3_ΔEnv contains a premature stop codon in the Env open reading frame (ORF) resulting from a 2-bp fill-in of an NdeI site. Plasmid pEnv-4059 encoding an R5-tropic Env from a clinical HIV-1 isolate was kindly provided by R. Swanstrom (University of North Carolina, Chapel Hill, NC, USA) [36]). Plasmid pVpr.IN.enhanced GFP (eGFP) encoding a Vpr.IN.eGFP fusion with a protease cleavage site between Vpr and IN was kindly provided by A. Cereseto (CIBIO, Mattareo, Italy [37]). Plasmid pMD2.G encoding the vesicular stomatitis virus glycoprotein (VSV-G) was generated by D. Trono (EPFL, Lausanne, Switzerland) and obtained through Addgene (#12259). Plasmid pcDNA3.1Zeo^+^ mammalian expression vector was obtained from Thermo Fisher Scientific (Waltham, MA, USA). The packaging plasmid containing the Vpx-interacting motif in group-specific antigen protein (Gag; pΔR8.9 NSDP) and the plasmid encoding Vpx_mac239_, pcDNA.Vpx_mac239_, were described previously [38,39].

### 2.3. Antisera and Reagents

All standard reagents, unless indicated otherwise, were purchased from common commercial sources. In-house rabbit and sheep polyclonal antisera against HIV-1 CA were raised against purified recombinant protein. Monoclonal anti-CA antibody from hybridoma cell line 183 clone H12-5C (MAK183) was obtained through the acquired immune deficiency syndrome (AIDS) Research and Reference Reagent Program, Division AIDS, NIAID, and purified in-house. Goat anti-rabbit immunoglobulin G (IgG; heavy and light chain (H + L)) conjugated to horseradish peroxidase was purchased from Dianova (Hamburg, Germany). Mouse monoclonal laminA/C antibody (sc-7292) was purchased from Santa Cruz (Heidelberg, Germany). Affinity purified anti-SAMHD1 antibody (12586-1-AP) was purchased from Proteintech Inc (Chicago, IL, USA). Mouse monoclonal anti-α-tubulin antibody (T5168) was purchased from Sigma-Aldrich. Mouse monoclonal anti-CD11b conjugated to phycoerythrin (PE) and mouse monoclonal anti-CD163 conjugated to fluorescein isothiocyanate (FITC) were purchased from Miltenyi Biotech. Alexa Fluor-labeled secondary antibodies were purchased from Thermo Scientific Laboratories. Secondary antibodies conjugated with infrared dye IRDye700/800 used for immunoblot were purchased from Rockland Antibodies and assays (Limerick, PA, USA) or from LI-COR (Lincoln, NB, USA). A 10 mM stock solution of raltegravir (RAL; AIDS Research and Reference Reagent Program, Division of AIDS, NIAID) was prepared in water and stored in aliquots at −20 °C. Additionally, 10 mM stock solutions of efavirenz (EFV; AIDS Research and Reference Reagent Program, Division of AIDS, NIAID), maraviroc (MVC; Sigma-Aldrich), and PF-3450074 (PF74; Sigma-Aldrich) were prepared in dimethyl sulfoxide (DMSO) and stored at −20 °C. Furthermore, 50 mM stock solutions of 2′-deoxyadenosine (dA; Sigma-Aldrich, D668), 2′-deoxythymidine (dT; Sigma-Aldrich, T1895), and 2′-deoxycytidine (dC; Sigma-Aldrich, D0776) were prepared in water and stored at −20 °C. A 50 mM stock solution of 2′-deoxyguanosine (dG; Sigma-Aldrich, D7145) was prepared in 1 M ammonium hydroxide and stored at −20 °C.

### 2.4. Virus Production and Characterization

Viral particles were produced in HEK293T cells transfected with 70 µg of plasmid DNA (per T 175 cm^2^ flask) using calcium phosphate, following standard procedures. Pseudotyped R5 tropic HIV-1 viral particles were generated by co-transfection of pNL4-3ΔEnv with pEnv-4059 at a molar ratio of 4.5:1. VSV-G pseudotyped particles were prepared using the same molar ratio but using pMD2.G instead of HIV-1 Env. For producing viral particles labeled with IN.eGFP, pNL4-3ΔEnv was co-transfected together with pEnv-4059 and pVpr.IN.eGFP at a molar ratio of 4.5:1:1. Lentiviral vectors lacking or carrying Vpx_mac239_ were produced by co-transfection of pcDNA3.1Zeo^+^ or pcDNA.Vpx_mac239_, respectively, with pMD2.G and pΔR8.9 NSDP at a molar ratio of 1.4:1.4:2.

Particle-containing supernatants were collected 48 h after transfection, filtered through 0.45-µm nitrocellulose filters, and concentrated by ultracentrifugation through a 20% (*w*/*w*) sucrose cushion (SW28 rotor, 28,000 rpm, 1.5 h). Particle pellets were gently resuspended in phosphate-buffered saline (PBS) containing 10% heat-inactivated FCS and 10 mM Hepes (pH 7.5), before being stored in aliquots at −80 °C. For ddPCR experiments, particle-containing supernatants were treated prior to ultracentrifugation with 15 U/mL DNAseI (Sigma-Aldrich) and 10 mM MgCl_2_ for 2.5 h at 37 °C. Virion-associated RT activity was determined by an SYBR Green-Based Product Enhanced Reverse Transcription Assay as a measure for particle amounts [40]. Concentrations of CA were determined by an in-house enzyme-linked immunosorbent assay (ELISA). For this purpose, 96-well ELISA plates were coated with purified MAK183 anti-CA antibody (diluted in PBS) overnight in a humid chamber. Following blocking with 10% FCS in PBS, plates were incubated with samples, previously diluted in 0.1% Tween/PBS, and incubated overnight in a humid chamber. Afterwards, an in-house polyclonal rabbit anti-CA antiserum diluted 1:1000 in 10% FCS/PBS/1% Tween was added, and antigen detection was performed by incubation with anti-rabbit IgG (H + L) secondary antibody conjugated with horseradish peroxidase. Tetramethylbenzidine (TMB; Thermo Fisher Scientific) was added as a substrate, and absorbance readings were performed at 450 nm to detect enzymatic activity. Purified recombinant HIV-1 CA of known concentration was used as a standard. Infectivity of HIV-1 particles was characterized by titration on TZM-bl indicator cells followed by detection of β-galactosidase-expressing cells at 48 h p.i., as previously described [34].

### 2.5. Virus Infection and Transduction, Click-Labeling, and Immunostaining

To visualize HIV-1 reverse-transcription and pre-integration complexes (RTC/PIC), MDMs were seeded in eight-well LabTek chamber slides (#155411, Thermo Scientific, Waltham, MA, USA) and infected with 100 ng of CA of HIV-1 (IN.eGFP) in complete RPMI containing 10 µM 5-ethynyl-2´-deoxyuridine (EdU; Thermo Scientific). At 24 h post infection (p.i.), 5 µM MVC (Sigma-Aldrich) was added together with 10 µM EdU in fresh RPMI 1640, and infection was continued at 37 °C for the time indicated in the description of the experiment. At the end of the incubation period, cells were washed with PBS and fixed in 4% paraformaldehyde (PFA; Electron Microscopy Sciences, Hatfield, PA, USA) for 15 min at room temperature. Subsequently, cells were washed and permeabilized in 0.5% (*v*/*v*) Triton X-100 for 15 min and washed again. Cells were washed twice with 3% bovine serum albumin (BSA; *w*/*v*, in PBS) and click-labeling was performed for 30 min at room temperature in the dark using the Click-iT EdU Alexa Fluor 647 Imaging Kit (Thermo Fisher Scientific), following the manufacturer’s instructions. Click-labeling mix was removed, and cells were washed three times. For immunostaining, cells were blocked in 3% BSA for 1 h, followed by incubation with the primary antibody diluted in PBS and 0.5% BSA for 1h at room temperature in the dark. Samples were then washed three times for 2 min with PBS, and then incubated with the corresponding secondary antibody in PBS/0.5% BSA for 1 h at room temperature. 

### 2.6. Infectivity Assays and Time-of-Addition Experiments

For assessment of productive infection, approximately 10,000 MDMs/well were seeded in black 96-well plates (Costar #3606). Cells were infected in triplicate or quadruplicate with 15 ng of CA of HIV-1_NL4-3_ pseudotyped with R5-tropic Env or VSV-G per well (an amount previously determined in infectivity experiments using a similar number of MDMs from various donors and different CA concentrations to be equivalent to a multiplicity of infection (MOI) of ~0.1 with some donor-to-donor variability). After 24 h, medium was replaced and 5 µM MVC (Sigma-Aldrich) was added to block further entry events. Then, 5 µM EFV, 5 µM RAL (AIDS Research and Reference Reagent Program, Division AIDS, NIAID), 2 µM PF74 (Sigma-Aldrich), or the equivalent volume of DMSO was added to the cultures at different time points, as indicated in the respective experiments. At 72 h p.i., medium was replaced, and inhibitor was added to samples from all time points, except for the DMSO control samples. Infection was stopped at day 6 p.i. by treatment with 4% PFA for 90 min, and newly synthesized Gag was detected by immunostaining using anti-CA (rabbit) antiserum; nuclei were counterstained with Hoechst (Thermo Fisher Scientific). Plates were imaged in a fully automated screening microscope, as explained below. For experiments involving Vpx-mediated SAMHD1 degradation, cells were transduced with virus-like particles (VLP) corresponding to 150 mU of RT lacking or carrying Vpx_mac239_ 12 h before HIV-1 infection. In experiments where extracellular nucleosides were added, medium supplemented with 100 µM of dA, dC, dG, and dT was added to the cells 2 h before infection.

### 2.7. Detection of HIV-1 RT Products with ddPCR

To detect HIV-1 RT products, MDMs were seeded in six-well plates and infected with 50 ng of CA of HIV-1_NL4-3_ pseudotyped with R5-tropic Env. For experiments involving Vpx-mediated SAMHD1 degradation, cells were transduced with 300–500 mU of RT VLP lacking or carrying Vpx_mac239_ 12 h before infection. Cells were harvested at different time points p.i. as indicated. For DNA extraction, cells were washed with PBS, detached from the plates, and spun down (5000 rpm, 5 min); cell pellets were lysed overnight at 55 °C using an in-house lysis buffer (10 mM Tris-HCl pH9.0, 0.1% Tritron X-100, 400 µg/mL proteinase K (Thermo Fisher Scientific), and double-distilled water (ddH_2_O)). Proteinase K was inactivated at 95 °C for 10 min, and lysates were stored at −20 °C or diluted and used for detection of RT products. Digital droplet PCR (ddPCR) was employed to detect HIV-1 RT products from infected cells, as described previously [41]. Early and late RT products were detected with two sets of primers/probe annealing to the 5′ long terminal repeat (LTR) or to the *gag* ORF of the genome, respectively. Additionally, another set of primers/probe to detect 2-LTR circles was used. The single-copy host gene encoding ribonuclease P protein subunit p30 (*RPP30*) was also quantified to normalize sample input (refer to Table 1 for sequences of primers and probes). Furthermore, 20-µL reactions containing 2–4 µL of diluted lysate, 900 nM each primer, 200 nM probe, 1× ddPCR Supermix for probes (no dUTP) (BioRad, Hercules, CA, USA), and water were prepared for each target and sample. Subsequently, droplets were generated using droplet generation oil for probes (BioRad), and immediately transferred to a 96-well microplate, as described previously [42]. PCR amplification was performed using the following program: initial denaturation and stabilization at 95 °C for 10 min, 40 cycles of denaturation at 94 °C for 30 s, and annealing/extension at 57 °C for 60 s, followed by 10 min at 98 °C. Subsequently, droplets were sorted and analyzed in a QX200 droplet reader (BioRad) using the software QuantaSoft v1.6 (BioRad) and the settings for absolute quantification. Results were analyzed using the same software, and copy numbers were normalized to the copy numbers of the housekeeping gene.

### 2.8. Flow Cytometry

To analyze macrophage differentiation and polarization, cells were washed and detached using StemPro Accutase (Thermo Fisher Scientific) for 30 min at 37 °C. Cells were then fixed in 4% PFA for 15 min, washed twice with PBS, and spun down. Pellets were resuspended in PBS and incubated with anti-CD11b-PE or anti-CD163-FITC for 1 h on ice, protected from light. Subsequently, cells were washed twice, and analyzed using an FACS Calibur flow cytometer (BD Biosciences, Franklin Lakes, NJ, USA). Unstained cells were used as a control for gating. Results were analyzed with the software FlowJo v10 (FlowJo, LLC, Ashland, OR, USA).

### 2.9. SDS-PAGE and Western Blot

Cells were washed with PBS and lysed in 3× Laemmli SDS buffer at 95 °C for 10 min. Lysates were separated by SDS polyacrylamide gel electrophoresis (PAGE) and proteins were transferred to a methanol-activated polyvinylidene fluoride (PVDF) membrane (Merck Millipore, Burlington, MA, USA). SAMHD1 and α-tubulin were detected by probing the membrane with anti-SAMHD1 and anti-α-tubulin antibodies, followed by secondary antibodies coupled to IRDye 700/800 (Rockland Antibodies, Limerick, PA, USA). Fluorescent signals were detected using a LI-COR Odyssey CLx scanning system. To quantify band intensities, blots were analyzed using the Odyssey Image Studio v5.2 software (LI-COR).

### 2.10. Microscopy

Three-dimensional (3D) image series were acquired with a Perkin Elmer Ultra VIEW VoX 3D spinning disk confocal microscope (SDCM) using a 100× oil immersion objective (numerical aperture (NA) 1.4) (Perkin Elmer, Waltham, MA, USA). Z-stacks with a spacing of 200 nm were acquired in the 405-, 488-, 561-, and 640-nm channels. For scoring infectivity, after immunostaining, plates were imaged using a high-throughput wide-field Olympus IX-81 inverted microscope with the 10× air immersion objective (NA 0.45). Images were recorded in the 4′,6-diamidino-2-phenylindole (DAPI) and Cy5 channels using the Scan^R/Xcellence software (Olympus, Tokyo, Japan). Sixteen positions were acquired per well.

### 2.11. Image Analysis

Analysis of individual HIV-1 RTC/PIC in 3D volumes of single infected cells was performed with Imaris 9.2 (Bitplane, Zürich, Switzerland). For this, images were deconvolved with Autoquant X3 (Media Cybernetics, Rockville, MD, USA) using constrained maximal likelihood estimation (CMLE) with 10 iterations and signal-to-noise ratio (SNR) = 20. 3D reconstructions of deconvolved images were done with Imaris, and IN.eGFP positive objects were identified using the spot detection function of the software. Camera offset was subtracted, and spots were detected using an estimated *XY* diameter of 300 nm, together with a quality filter detecting the intensities at the center of the spot. Mean signal intensities in the four recorded channels were measured.

To detect and quantify infected cells in images acquired with the automated system, we used a previously published script in MatLab (MathWorks [46]). Briefly, objects were identified using the Hoechst signals, and the difference in signal intensities between background and signals from actual nuclei was used to generate a nuclear mask. This mask was used to segment the cytoplasm, and CA signal intensities from the whole cell were measured. Mock-infected samples were used as a reference to set an intensity threshold to distinguish between non-infected and infected cells.

## 3. Results

### 3.1. Early HIV-1 Replication Is Slow in Primary Macrophages

In order to study HIV-1 infection in primary human MDMs, terminally differentiated macrophages were prepared from primary monocytes from healthy blood donors. Various protocols for macrophage differentiation are used in the field, resulting in differently activated cells. Macrophage populations are classified based on their immune activation status and cytokine production into M0 (non-polarized), M1, and M2 cells [47]. It was reported that addition of certain cytokines or growth/differentiation factors such as M-CSF (commonly associated with M2 macrophages) or GM-CSF (commonly associated with M1 macrophages) before or after infection may affect HIV-1 replication efficiency [14,15]. We, therefore, analyzed whether macrophage differentiation into M0, M1, or M2 populations affected HIV-1 infectivity. Monocytes were isolated from peripheral blood mononuclear cells (PBMC) and differentiated into M0, M1, or M2a macrophages by the addition of human AB serum, GM-CSF and IFNγ, or M-CSF and IL-4, respectively. The differentiation status of the resulting cell populations was verified by determining surface levels of CD11b and CD163 using flow cytometry [48] (Figure S1A, Supplementary Materials). Subsequently, cells were infected with HIV-1_NL4-3_-derived particles pseudotyped with an R5-tropic envelope (Env) protein from a patient isolate [36]. At 24 h post infection (p.i.), medium was removed and 5 µM of the HIV-1 entry inhibitor maraviroc (MVC) was added to prevent further entry. At day 6 p.i., productive infection was assessed by detection of newly synthesized viral protein using an antiserum against CA. In agreement with previous reports [15,49], M1 macrophages were not efficiently infected; less than 3% of cells were scored as CA-positive. Efficiency of HIV-1 infection in M0 and M2a macrophages was comparable, with ~8% CA-positive cells detected in both cases (Figure S1B, Supplementary Materials). The following experiments were performed in non-polarized (M0) primary macrophages generated from monocytes by stimulation with human AB serum (termed MDM throughout the report).

**Figure 1:**
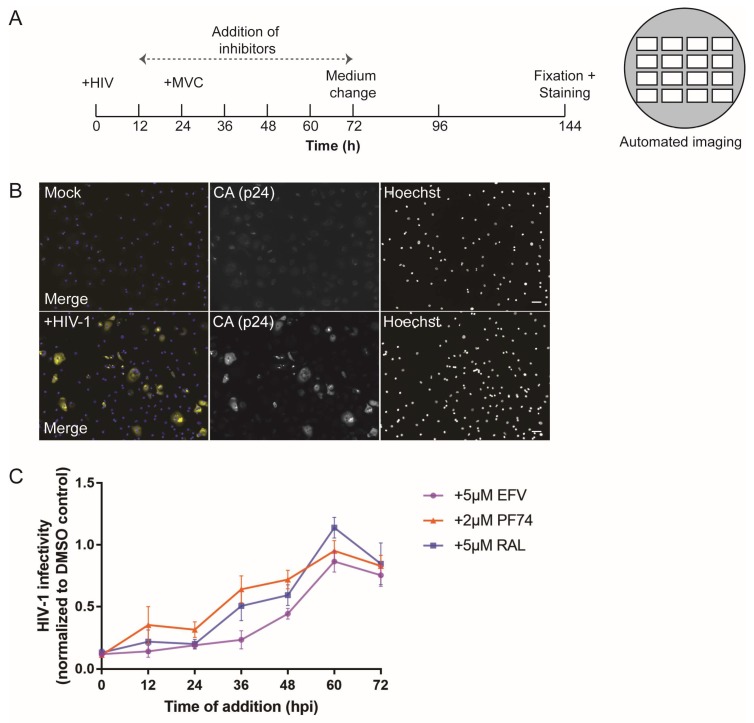
Time course analysis of human immunodeficiency virus type 1 (HIV-1) early post-entry events in primary macrophages. (**A**) Scheme of inhibitor time-of-addition experiments. (**B**,**C**) Monocyte-derived macrophages (MDMs) from six donors in three independent experiments were infected with R5-pseudotyped HIV-1_NL4-3_. At 24 h post infection (p.i.), medium was changed and 5 µM maraviroc (MVC) was added to prevent further entry events. At day 6 p.i., samples were fixed and stained. (**B**) Productive infection was assessed by immunostaining with anti-p24 capsid (CA) antiserum (yellow) and counterstaining with Hoechst (blue). Samples were imaged in an automated screening microscope. Scale bars represent 50 µm. (**C**) Solutions of 5 µM efavirenz (EFV; purple line), 5 µM raltegravir (RAL; blue line), or 2 µM PF74 (orange line) were added at the indicated time points to different samples. At 72 h p.i., medium was replaced in all samples by fresh medium containing the respective inhibitors and infection was again scored at day 6 p.i. The mean proportion of infected cells from triplicate samples of all donors in treated samples normalized to the respective dimethyl sulfoxide (DMSO) control is shown. Error bars represent standard error of the mean (SEM). Refer to Figure S1 (Supplementary Materials) to see the results for individual donors.

In order to obtain a detailed timeline for post-entry steps, we performed single-round infection and inhibitor time-of-addition experiments (Figure 1). MDMs were infected with R5-pseudotyped HIV-1_NL4-3_. Pseudotyping ensured that infection was restricted to a single round as newly synthesized particles lacked the viral Env proteins. At 24 h p.i., the tissue culture medium was changed and 5 µM MVC was added to prevent further entry events. Parallel cultures were then treated with specific HIV-1 inhibitors targeting either reverse transcription (efavirenz, EFV) or genome integration (raltegravir, RAL). The small CA-binding molecule PF74 was used as an inhibitor of nuclear import. While high concentrations of this compound (>10 µM) can impair reverse transcription, presumably by affecting capsid stability, the lower concentration (2 µM) of PF74 used in our experiments was found to block nuclear entry of subviral complexes without affecting reverse transcription [50,51,52]. The different inhibitors were added at various time points post infection, spanning a period from 12 h to 72 h p.i. (Figure 1A). Incubation of all samples was then continued for a further 72 h in the presence of the respective inhibitor to ensure maximum HIV-1 antigen expression. Productive infection was assessed at day 6 p.i. by immunofluorescence detection of newly synthesized Gag, using an antiserum against the CA domain of the polyprotein.

As shown in Figure 1B, productively infected cells were clearly distinguishable from non-infected cells (identified by Hoechst stain) based on CA signal intensities. An automated microscopic set-up and data analysis pipeline was used to determine the proportion of cells infected under each condition. The values obtained under inhibitory conditions were normalized to the proportion of infected cells observed upon DMSO treatment to determine the resilience to the drugs at each time point of addition (Figure 1C). Unexpectedly, loss of sensitivity to inhibitors of reverse transcription and integration was largely parallel in these experiments. Although some donor-to-donor variation was observed (Figure S1C, Supplementary Materials), very low levels of infection were detected when these inhibitors were added in the first 24 h of infection. Roughly 50% of resistance was observed at 48 h p.i., and the inhibitors showed no significant effect at 60 h p.i. or later, indicating that reverse transcription, nuclear import, and viral cDNA integration into the host cell genome were all largely completed at this time. The curve was similar for PF74; however, in this case, some resilience was observed already at 12 h p.i. (Figure 1C). Taken together, these experiments indicate a lag time of 24–36 h during which completion of HIV-1 reverse transcription and/or integration is not detectable in MDMs, and show that post-entry events are completed between 48 h and 60 h post infection in these cells.

**Figure 2:**
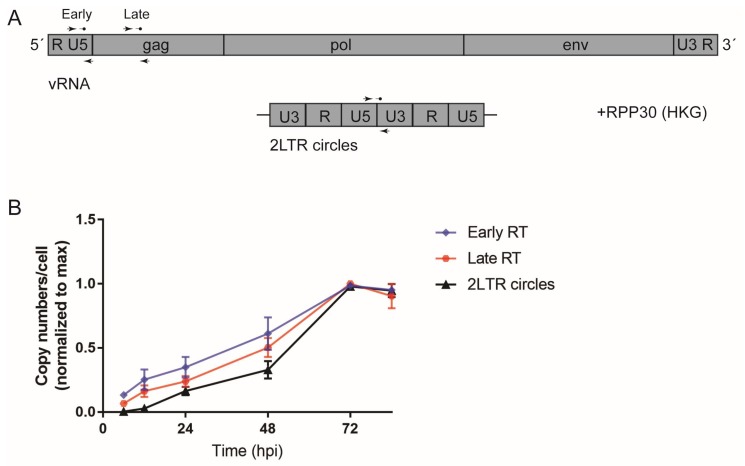
Quantitation of reverse transcriptase (RT) products using digital droplet PCR (ddPCR). MDMs from seven donors in four independent experiments were infected with R5-pseudotyped HIV-1_NL4-3_. At 24 h p.i., medium was changed and 5 µM MVC was added. Cells were harvested at the indicated time points and HIV-1 RT products were quantitated in cell lysates by ddPCR as detailed in Section 2.7. (**A**) Scheme of the binding sites of the set of primers used for detection of early and late RT products as well as 2-long terminal repeat (LTR) circles. Cellular *RPP30* was used as a housekeeping gene for normalization. (**B**) Copy numbers of early (5′-LTR; blue line) and late RT (*Gag*; red line) products or 2-LTR circles (black line) were normalized to the copy numbers of the housekeeping gene at different time points p.i. for each donor, and these values were subsequently normalized to the highest absolute number detected. Each symbol represents the mean of all the donors. Error bars represent SEM. Refer to Figure S2 (Supplementary Materials) to see individual infectivity data for all donors in the parallel experiment.

The lack of an apparent lag between completion of reverse transcription and integration suggested that completion of reverse transcription and/or nuclear entry represent rate-limiting steps of HIV-1 replication in MDMs. In order to study kinetics of reverse transcription in more detail, RT products from infected cells were quantitated at different time points p.i. using sensitive digital droplet PCR (ddPCR) [41]. MDMs from different donors were infected with R5-pseudotyped HIV-1 and harvested at various time points over a period of four days. Cell lysates were subjected to ddPCR using specific primer combinations to detect early and late products of reverse transcription as well as 2-LTR circles as a surrogate marker for viral cDNA nuclear import [53] (Figure 2A). Productive infection was scored in parallel samples by immunofluorescence as before. Absolute copy numbers of all targets positively correlated with the infection efficiency scored for the respective donor (Figure S2A, Supplementary Materials). The peak of early RT product copy numbers was detected between 60 h and 72 h p.i. After this time, copy numbers reached a plateau or dropped (Figure S2B, Supplementary Materials; Figure 2B), suggesting degradation of non-integrated transcripts. Late RT products peaked at 72 h p.i., followed by a plateau phase or decline (Figure S2B, Supplementary Materials; Figure 2B). Copy numbers determined for 2-LTR circles were significantly less abundant and peaked at the same time point as late RT products (Figure S2C, Supplementary Materials; Figure 2B). These results indicate that reverse transcription is slow in macrophages compared to the time frame reported for lymphocytes [17] and corroborate our initial conclusions from the time-of-addition experiments.

### 3.2. Visualization of HIV-1 RTC/PIC Revealed Timeframe of Nuclear Translocation

The bulk analyses described above indicated that HIV-1 post-entry events in primary macrophages occur slowly over a 2–3 day time course, and that completion of reverse transcription and/or nuclear import of the viral genome (as assessed by the indirect readout of 2-LTR circle formation) are rate-limiting steps in this process. For a more detailed characterization of the time course of RTC/PIC nuclear import, we proceeded with direct visualization of RTC/PIC appearance and intracellular distribution over the course of the infection cycle. We previously established a microscopy-based approach, which allows us to detect individual active HIV-1 RTC/PIC within infected cells [50]. For this, RTC/PIC are marked by incorporation of the key protein component integrase (IN) fused to eGFP [37] into the infecting virion. In order to identify productive subviral complexes within newly infected cells, nascent viral cDNA is tagged by incorporation of the thymidine analog 5-ethynyl-2′-deoxyuridine (EdU), which can in turn be detected via click-labeling with fluorophores. Active RTC/PIC are then identified in confocal micrographs based on the co-localization of EdU click-label and IN.eGFP signals [50]. Nuclear PIC positive for EdU and IN.eGFP may represent unintegrated or integrated proviral complexes.

**Figure 3:**
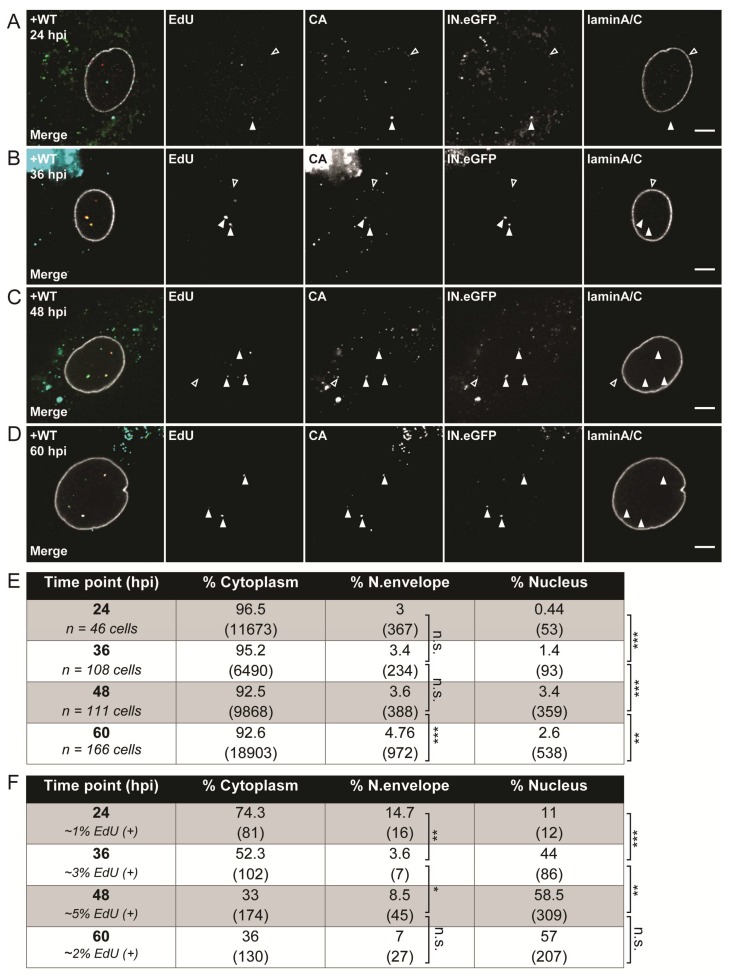
Subcellular detection of HIV-1 reverse-transcription and pre-integration complexes (RTC/PIC). MDMs from four donors in three independent experiments were infected with 100 ng of CA of R5-pseudotyped HIV-1_NL4-3_ (IN.eGFP) in the presence of 10 µM 5-ethynyl-2′-deoxyuridine (EdU). At 24 h p.i., medium was changed and further viral entry was blocked by 5 µM MVC. Cells were fixed at 24 (**A**), 36 (**B**), 48 (**C**), and 60 h p.i. (**D**), click-labeled, and immunostained with anti-CA antiserum (cyan); the nuclear envelope was marked by immunostaining with anti-laminA/C antibody (white). EdU and IN.eGFP signals in the merged panels are represented in red and green, respectively. Exemplary complexes of different types are indicated by arrowheads: solid arrows indicate co-localizing EdU and IN.eGFP signals; empty arrows identify HIV-1 complexes lacking EdU signals. Scale bars represent 5 µm. (**E**) Proportion of IN.eGFP-positive objects in *n* cells in three subcellular localizations at different time points. Numbers of detected objects are given in parentheses. Proportions were compared using a two-tailed *Z*-test (α = 0.05); *** *p* < 0.0001; ** *p* < 0.002; n.s.: not significant. (**F**) Proportion of IN.eGFP- and EdU-positive objects from (**E**) in *n* cells in three subcellular localizations at different time points. Numbers of detected objects are given in parentheses. Proportions were compared using a two-tailed *Z*-test (α = 0.05); *** *p* < 0.0001; ** *p* < 0.005; * *p* = 0.022; n.s.: not significant.

MDMs from different donors were infected with IN.eGFP-labeled R5-pseudotyped HIV-1_NL4-3_ in the presence of 10 µM EdU. At 24 h, medium was changed and 5 µM MVC was added to prevent further infection. Cells were fixed at various time points p.i. and click-labeled. Since we previously observed CA associated with both cytoplasmic and nuclear RTC/PIC in MDMs [50], samples were also stained with antibodies against CA. Anti-laminA/C immunostaining was performed to mark the nuclear envelope. Cells were imaged by confocal microscopy and 3D volumes were reconstructed. RTC/PIC detected were classified according to intracellular localization as (i) cytoplasmic; (ii) close to the nuclear envelope; or (iii) within the nucleus (Figure 3A–D). Relative proportions of RTC/PIC at each of these localizations were quantitated based on 3D reconstructions of cells fixed at different time points p.i. These results are summarized in Figure 3E,F.

Only a minor proportion of all complexes detected were found to be EdU-positive at all times (from ~1% at 24 h to a maximum of ~5% at 48 h p.i.), and the vast majority of IN.eGFP-positive objects (>90%) were localized in the cytoplasm at all time points (Figure 3E,F). This may reflect endosomal uptake without fusion and/or abortive entry without successful reverse transcription. About 3% of all viral complexes were detected in close vicinity to the nuclear envelope already at the earliest observation time point (24 h p.i.; Figure 3A,E), with a similar proportion detected at the subsequent observation times. In contrast, only few objects (0.44% of total) were found within the nucleus at 24 h p.i., and this proportion significantly increased over the following days to ~1.4% at 36 h (Figure 3B,E) and a maximum of ~3% at 48 h (Figure 3C,E).

When focusing exclusively on productive RTC/PIC (as defined by a positive EdU signal co-localizing with IN.eGFP; see examples in Figure 3F), we observed a generally similar time course, consistent with previous reports indicating that reverse transcription is not required for nuclear import [54,55]. However, the relative proportion of productive nuclear PIC was significantly higher at all time points analyzed. At 24 h p.i., 15% of all EdU-positive complexes were observed in the vicinity of the nuclear envelope, and this number remained relatively stable over the rest of the observation period. Roughly 10% of productive complexes were already inside the nucleus at 24 h p.i., while this proportion was <1% for all INeGFP-positive objects (Figure 3E,F). The proportion of nuclear EdU-positive PIC increased to 40% and >50% at 36 h and 48 h p.i., respectively, with no further changes observed at 60 h p.i. (Figure 3D,F). In agreement with our previous observations in macrophages [50], the vast majority (>90%) of productive nuclear PIC were found to be clearly CA-positive, with no apparent difference between time points.

The relative fluorescence intensity of individual particles in the EdU channel is proportional to the amount of EdU incorporated by the viral RT and, thereby, provides information on the progression of reverse transcription (although a direct quantitative correlation between fluorescence intensity and length of the nucleic acid is yet to be established). We, therefore, compared the distribution of signal intensities of EdU-positive complexes detected at 48 h p.i. at the different subcellular localizations (Figure S3, Supplementary Materials). In all cases, we observed a clear peak population with a comparatively low signal intensity of 5000–10,000 a.u. over background; however, complexes with considerably higher intensity were also detected. In the cytoplasm, complexes with intensities above 10,000 a.u. amounted to about 20%, with peak values up to ~100,000 a.u. and a mean signal intensity of the whole population of ~8900 a.u. Complexes detected in the nucleus were clearly brighter on average, with almost half of all complexes (~47%) displaying intensities >10,000 a.u., with maximum intensities up to ~300,000 a.u. and a total mean intensity of 24,800 a.u. Whether this apparent shift to higher intensity in the nucleus indicates that nuclear import is influenced by the progression of reverse transcription, or that HIV-1 reverse transcription in MDMs is only completed in the nucleus cannot be inferred from bulk time-lapse experiments and remains to be determined. Intriguingly, complexes detected close to the nuclear envelope did not exhibit an intermediate phenotype, but fell almost exclusively (95%) into the low-EdU-intensity class of <10,000 a.u., with a population mean of 4800 a.u.

### 3.3. Type and Time of Cell Entry Does Not Affect Replication Kinetics in Macrophages

**Figure 4:**
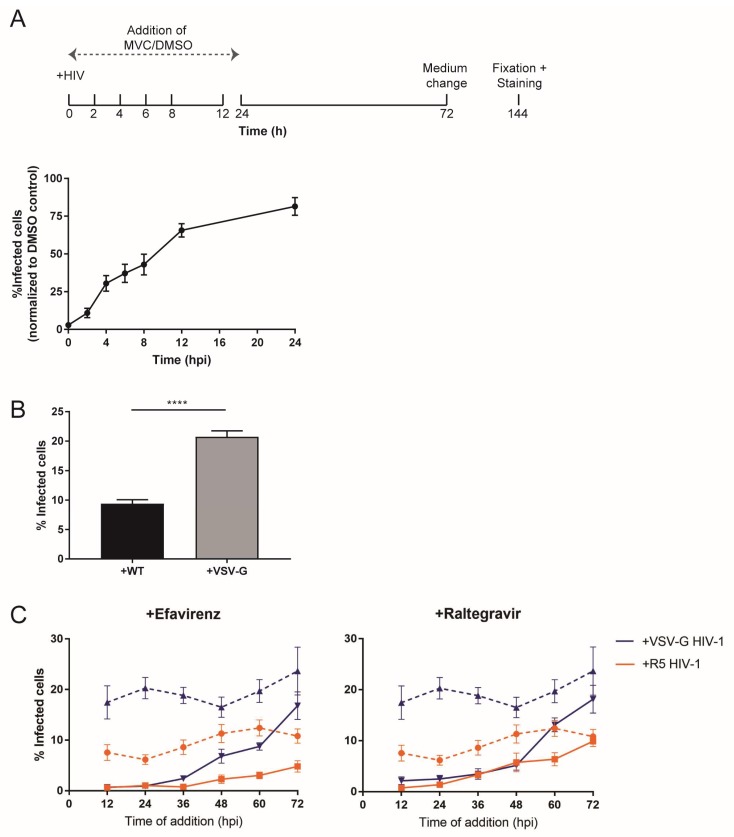
Effect of time and type of cell entry on HIV-1 replication kinetics. (**A**) MDMs from four donors were infected with R5-pseudotyped HIV-1_NL4-3_. Subsequently, 5 µM MVC or an equivalent volume of DMSO was added to the medium at the indicated time points p.i. Medium was replaced, and infection was continued in the presence of the inhibitor until day 6. Cells were fixed, and productive infection was scored by CA immunostaining. Proportions of infected cells were normalized to the highest proportion observed in the DMSO control. The graph shows mean values and SEM from triplicates or quadruplicates of all donors after normalization. (**B**,**C**) Influence of entry route: (**B**) MDMs from four donors were infected with R5- or vesicular stomatitis virus (VSV-G) pseudotyped HIV-1_NL4-3_. At 24 h p.i., medium was changed, and infection continued until day 6 p.i. Cells were fixed, and infectivity was scored as above. The graph shows mean values and SEM from the four samples. Statistical significance was assessed with a non-paired two-tailed Mann–Whitney test; **** *p* < 0.0001. (**C**) MDMs from the same donors as in (**B**) were infected with equal amounts of R5-pseudotyped (orange lines) or VSV-G pseudotyped (blue lines) HIV-1_NL4-3_. After 24 h, medium was changed. Solutions of 5 µM EFV (left plot, solid lines) or 5 µM RAL (right plot, solid lines), or DMSO (dashed lines) were added every 12 h until 72 h p.i. At this time point, medium was changed, and infection continued until day 6 in the presence of the inhibitors. Infectivity was scored as before. Mean values from infectivity values of the replicates of all donors and SEM are shown.

Many studies addressing HIV-1 replication in macrophages employed pseudotyping of virus particles with the glycoprotein of vesicular stomatitis virus (VSV-G) in order to increase infection efficiency (e.g., [31,56,57]). In addition to enhancing entry efficiency, this strategy also results in redirection of the viral entry pathway exclusively to clathrin-mediated endocytosis [58,59]. Since an influence of the entry route on post-entry events is conceivable [59], we were interested in determining the effect of this alteration on the time course of post-entry events. As a baseline, we analyzed kinetics of entry mediated by the R5-tropic HIV-1 envelope in an inhibitor time-of-addition experiment. The co-receptor antagonist MVC was added to MDM at different time points after infection, and infectivity was scored by CA immunostaining at day 6 p.i. as in the previous experiments (Figure 4A). At 4 h p.i., resistance to the addition of MVC was around 30% and a large fraction of viral particles were still blocked, in contrast to T-cell lines, where >50% of cell entry events occurred within the first 2–4 h of infection [6,7]. Resilience to the inhibitory effects of MVC was around 50% at 8 h p.i. with some variation between donors, and reached near completion at 24 h p.i. (Figure 4A), indicating that the half-time for productive entry into primary macrophages is ca. 8 h.

To analyze the effect of the entry pathway on post-entry dynamics, MDMs were infected in parallel with HIV-1 particles pseudotyped with either the R5-tropic HIV-1 Env or VSV-G, and cultures were treated with EFV or RAL at different time points as above. Productive infection was again scored at day 6 p.i. In line with previous reports [31,57], we observed that overall infection efficiency in MDMs was enhanced roughly twofold upon pseudotyping with VSV-G (Figure 4B). The relative time course of post-entry events as assessed by the acquisition of resilience toward RT or IN inhibitors, however, was very similar for both viruses (Figure 4C). These findings argue against the route and efficiency of cell entry being determinants of the slow post-entry dynamics in MDMs.

### 3.4. Low Availability of dNTPs Restricts HIV-1 Replication in MDMs

A low cytoplasmic dNTP concentration in macrophages (~130–250-fold lower than in resting or activated CD4^+^ T cells [21]) was proposed to be a major obstacle to productive HIV-1 infection [60]. The triphosphohydrolase activity of the host protein SAMHD1 is important for maintaining low dNTP levels in macrophages, and the protein was recognized as an important host cell restriction factor of HIV-1 infection [23]. HIV-2 and SIV, but not HIV-1, express a protein termed viral protein X (Vpx), which is able to overcome this restriction by mediating proteasomal degradation of human SAMHD1 [29,30,61].

We assessed the effect of increasing intracellular dNTP levels on HIV-1 post-entry dynamics in MDMs. For this, we followed two different approaches. In one set of samples, we supplemented the tissue culture medium before and during infection with a high concentration of deoxynucleosides as precursors for dNTP synthesis, as done previously [62]. In another set of experiments, we transduced host cells with lentiviral vector particles carrying exogenously expressed Vpx from SIVmac239 before infection, and compared the time course of post-entry events with that in cells pre-treated with control particles lacking Vpx.

**Figure 5:**
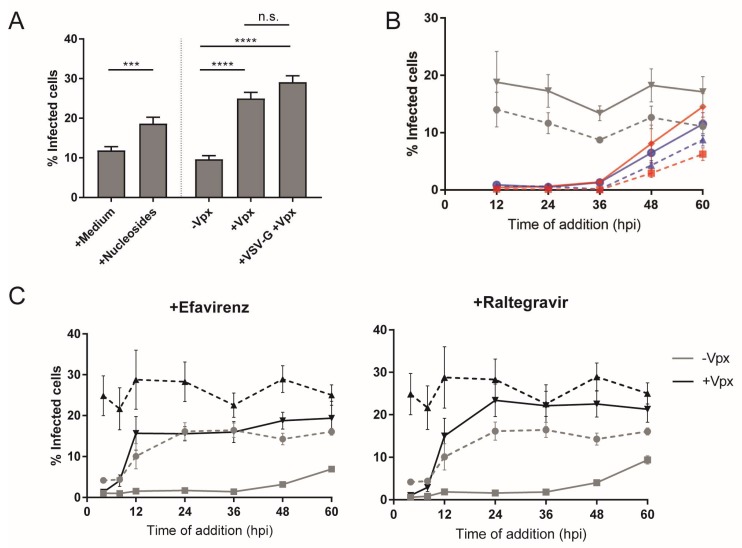
Effect of deoxynucleoside addition or viral protein X (Vpx_mac239_)-mediated sterile alpha motif (SAM) and histidine/aspartate (HD) domain-containing protein 1 (SAMHD1) degradation on early HIV-1 replication. (**A**) MDMs were either pre-incubated or not with nucleosides (100 µM each) 2 h prior to infection or transduced with HIV-1 virus-like particles (VLPs) lacking or carrying Vpx_mac239_ 12 h prior to infection. Subsequently, cells were infected with R5- or VSV-G pseudotyped HIV-1_NL4-3_. Infectivity was scored at day 6 p.i. by CA immunostaining. Error bars represent SEM of infection rates from all the replicates of all the donors. Statistical significance was assessed by a non-paired two-tailed Mann–Whitney test; **** *p* < 0.0001; *** *p* = 0.0001; n.s.: not significant. (**B**) MDMs from four donors in two independent experiments were pre-incubated for 2 h in medium (dashed lines), or medium supplemented with 100 µM deoxynucleosides (solid lines) and then infected with R5-pseudotyped HIV-1_NL4-3_. At 24 h p.i., medium was changed and 5 µM MVC was added. Solutions of 5 µM EFV (red lines) or 5 µM RAL (blue lines), or the equivalent volume of DMSO (gray lines) were added to the cultures at the indicated time points and infectivity was scored at day 6 p.i. The graph summarizes mean infectivity values from four donors, each measured in quadruplicate. Error bars represent SEM. (**C**) MDMs from four donors in two independent experiments were transduced with HIV-1 VLPs lacking (grey lines) or carrying (black lines) Vpx_mac239_. After 12 h, medium was changed, and cells were infected in quadruplicates with R5-pseudotyped HIV-1_NL4-3_. Solutions of 5 µM EFV (left, solid lines), RAL (right, solid lines), or DMSO (dashed lines) were added at the indicated time points. At day 6 p.i., the proportion of infected cells was scored by CA immunostaining. The graph summarizes mean values from four donors, each measured in quadruplicate. Error bars represent SEM. The unexpected effect of the addition of DMSO to the cultures in the first time points under native conditions (−Vpx) was most likely caused by technical errors at the moment of infection.

Firstly, we performed an inhibitor time-of-addition experiment in infected cells cultured in the presence or absence of nucleosides added to the medium (100 µM each of all four deoxynucleosides added 2 h before infection; Figure 5A, left bars, and Figure 5B). Nucleoside addition increased overall infection efficiency of MDM nearly twofold compared to infection in the absence of deoxynucleosides (Figure 5A, left). The time-of-addition experiment showed full sensitivity toward inhibition of reverse transcription or integration for the initial 24–36 h p.i. under both conditions (Figure 5B). The number of cells refractory to the inhibitors increased more rapidly in the presence of nucleosides than in the control samples after this time (Figure 5B), reflecting the higher number of productively infected cells and suggesting enhancement of reverse transcription activity. Again, the timeline of resilience against EFV or RAL was similar in this experiment, suggesting that reverse transcription/nuclear import still represent a bottleneck under these conditions (Figure 5B).

In a second set of experiments, MDMs were transduced with lentiviral vector particles either lacking or carrying Vpx_mac239_ 12 h before infection. Degradation of SAMHD1 was monitored by immunofluorescence to determine the optimal time course and validate the decrease in SAMHD1 levels. At 12 h p.i., >90% of the cells exposed to the Vpx-carrying particles lacked a detectable SAMHD1 signal (Figure S4A, Supplementary Materials); these conditions were, thus, chosen for subsequent infection. In agreement with previous reports (e.g., [23,24,25,29]), cells that underwent SAMHD1 degradation displayed significantly higher infectability (ca. threefold increase) than cells treated with control particles. This effect was slightly, but not significantly, enhanced further when infection was performed with VSV-G pseudotyped virus (Figure 5A, right panel). Inhibitor time-of-addition experiments using EFV or RAL (Figure 5C) revealed similar time courses for MDMs transduced with particles lacking Vpx as observed in our previous experiments (compare Figure 1C). Again, overall infection levels were higher in cells pre-exposed to Vpx_mac239_, as evident from the higher numbers of infected cells observed in the DMSO control samples (dotted lines). Early replication events appeared to occur much faster in cells pre-exposed to Vpx_mac239_ compared to control transductions. While, at the earliest time points analyzed (up to 8 h p.i.), the difference between Vpx_mac239_-treated cells and control cells was minor, pronounced differences were observed at the later time points. At 12 h p.i., the majority of Vpx_mac239_-exposed cells were refractory to the effect of both drugs, indicating that reverse transcription and integration were already completed in a large proportion of infected cells at this time. After 24 h p.i., resilience in these cells reached nearly 100%. In contrast, cells treated with particles lacking Vpx remained fully sensitive to both drugs up to ~36 h p.i. In summary, these results corroborate that transduction of Vpx alleviates restriction to HIV-1 infection in macrophages and show that this is reflected in the kinetics of early viral replication.

**Figure 6:**
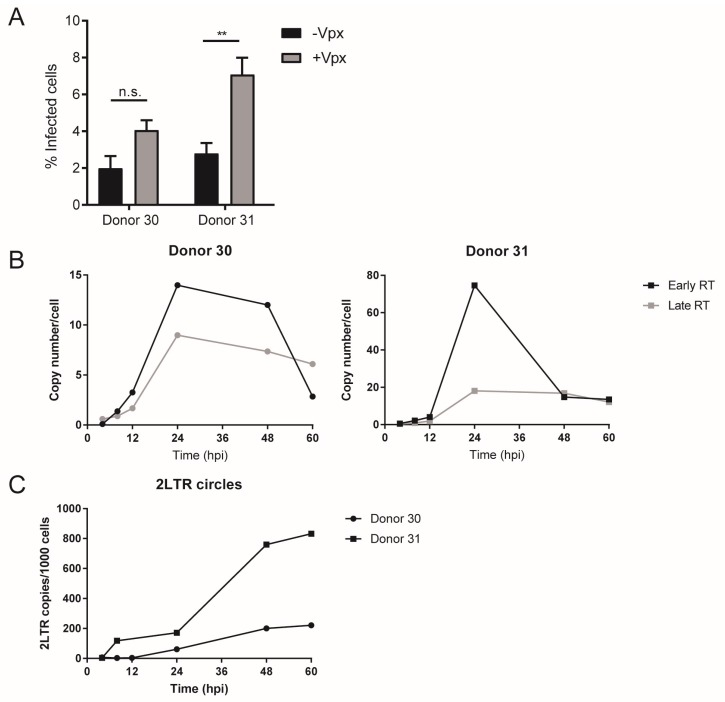
Influence of SAMHD1 degradation on reverse transcription efficiency. MDMs from two donors were transduced with VLPs lacking or carrying Vpx_mac239_. After 12 h, cells were infected with R5-pseudotyped HIV-1_NL4-3_. At 24 h p.i., medium was replaced and 5 µM MVC was added. (**A**) At day 6 p.i., infected cells were scored by CA immunostaining. Mean values and SD from quadruplicate samples are shown. Statistical significance was assessed by a non-paired two-tailed Mann–Whitney test (donor 30) or a non-paired two-tailed Student′s *t*-test (donor 31); ** *p* = 0.0018; n.s.: not significant. (**B**,**C**) Cells infected with VLPs carrying Vpx_mac239_ cells were lysed at the indicated time points and RT products were detected by ddPCR as described in Section 2.7. Absolute copy numbers of early (black lines) and late RT (gray lines) products, and 2-LTR circles (**C**) at each time point p.i. were normalized to the copy numbers of the housekeeping gene. Each symbol shape represents a donor.

In order to characterize the effect of Vpx-mediated SAMHD1 degradation on the dynamics of reverse transcription in more detail, we quantitated different RT products in samples from inhibitor time-of-addition experiments by ddPCR, including early time points post infection. MDMs were transduced with VLP lacking or carrying Vpx_mac239_ for 12 h. SAMHD1 depletion was validated by immunoblot (Figure S4B, Supplementary Materials) and only samples from the two donors showing a strong reduction of SAMHD1 levels upon Vpx_mac239_ transduction (>70% reduction of bulk protein levels) were included in the analyses. Overall infection efficiency was very low for these donors (~2–3% infected cells); however, in agreement with our previous observations, transduction of Vpx_mac239_ resulted in a nearly twofold increase of HIV-1 infection (Figure 6A).

Copy numbers of early RT products did not only peak earlier upon prior transduction with Vpx_mac239_-carrying particles (24 h vs. 48 h p.i.), but also reached higher levels than in cells transduced with particles lacking Vpx (Figure 6B; Figure S5A, Supplementary Materials), suggesting that SAMHD1 degradation results in both more rapid and more efficient reverse transcription. Late RT products peaked at 24 h p.i. in the presence of Vpx_mac239_ in the host cells (vs. 72 h p.i. in its absence), and again were significantly higher (~3–20-fold higher) than in cells transduced with VLP lacking Vpx (Figure 6B; Figure S5A, Supplementary Materials). Levels of 2-LTR circles were very low in control cells in accordance with the low infection rates observed, and peaked at time points from 48–60 h dependent on the donor, as previously observed (Figure S5B, Supplementary Materials). Exposure to Vpx_mac239_ enhanced the levels of 2-LTR circles detected; these replication byproducts continued increasing over the whole observation time in the individual donors, suggesting that genome circularization of non-productive complexes is slow compared to the enhanced rate of nuclear entry (Figure 6C).

**Figure 7:**
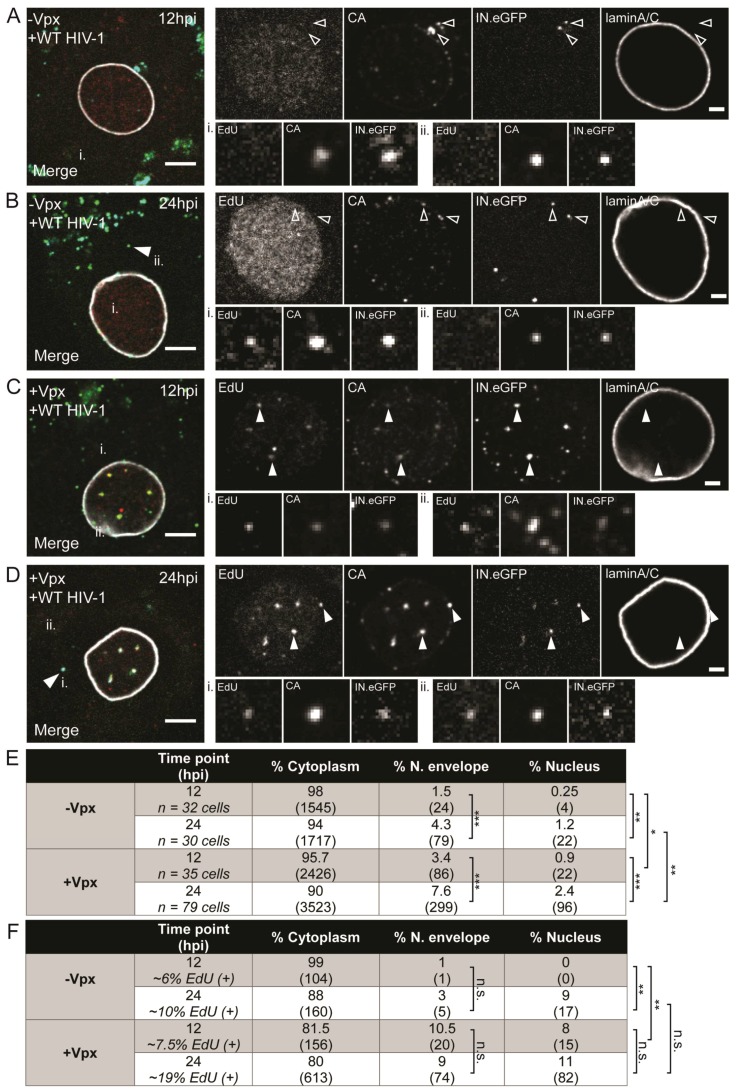
Detection of nascent viral complementary DNA (cDNA) by EdU click-labeling following SAMHD1 degradation. MDMs from three donors were transduced with 150 mU of RT of VLPs lacking (**A**,**B**) or carrying Vpx_mac239_ (**C**,**D**). After 12 h, cells were infected with R5-pseudotyped HIV-1_NL4-3_ (IN.eGFP) in the presence of 10 µM EdU. Cells were fixed at 12 (**A**,**C**) or 24 h p.i. (**B**,**D**) and click-labeled as described in Section 2.5. CA (cyan) and laminA/C (white; labeling the nuclear envelope) were detected by immunostaining; EdU and IN.eGFP signals in the overlays are represented in red and green, respectively. Enlargements of the nuclear region are displayed in the top right panels. Examples of individual RTC/PIC (**i**,**ii**) indicated in the merged panels are enlarged in the lower right. Note that focal planes may differ between overview of the nuclear region and particle enlargement. Exemplary complexes of different types are indicated by arrowheads: solid arrows indicate co-localizing EdU and IN.eGFP signals; empty arrows identify HIV-1 complexes lacking EdU signals. Scale bars represent 5 µm (overview) or 2 µm (enlargements). (**E**) Proportion of IN.eGFP-positive objects in *n* cells in three subcellular localizations at the indicated time points in cells treated with plasmid cDNA or Vpx VLPs. Numbers of detected objects are given in parentheses. Proportions were compared using a two-tailed *Z*-test (α = 0.05); *** *p* < 0.0001; ** *p* < 0.002; * *p* = 0.01; n.s.: not significant. (**F**) Proportion of IN.eGFP- and EdU-positive objects from (**E**) in *n* cells in three subcellular localizations at different time points in the presence or absence of Vpx_mac239_. Numbers of detected objects are given in parentheses. Proportions were compared using a two-tailed *Z*-test (α = 0.05); *** *p* < 0.0001; ** *p* < 0.005; * *p* = 0.01; n.s.: not significant.

To complement these results, we performed microscopic analyses of subviral complexes in cells pre-treated with particles lacking or carrying Vpx_mac239_ at early time points p.i. Transduced cells were infected with virus carrying IN.eGFP and subjected to EdU click-labeling for detection of nascent viral cDNA. Cells were fixed at 12 and 24 h p.i., click-labeled, and immunostained with anti-CA and anti-laminA/C antibodies. As observed in our previous experiments (compare Figure 3), the vast majority of IN.eGFP-positive objects (>90%) were localized in the cytoplasm at both time points. Proportions of IN.eGFP-positive objects in the vicinity of the nuclear envelope and within the nucleus increased from 12 h to 24 h p.i. under both conditions (Figure 7A–E), but values were 2–3-fold higher in all cases for the cells pre-exposed to Vpx_mac239_ compared to the corresponding value for the control cells, indicating more rapid transport toward and into the nucleus. In agreement with our observation that levels of early and late RT products at 24 h p.i. were greatly increased upon exposure of cells to Vpx_mac239_, a higher proportion of IN.eGFP-positive objects displayed detectable EdU signals at this time point (~20% vs. ~10% for cells treated with control particles; Figure 7F).

Again, only a minor proportion of objects in the cytoplasm or near the nuclear envelope, but the majority of nuclear complexes (70–80%), were EdU-positive in all samples. While we refrained from a detailed analysis of EdU signal intensity distributions because of the relatively low total number of EdU-positive complexes detected in these experiments, increased reverse transcription upon Vpx_mac239_ exposure was also reflected in the higher mean EdU signal intensities observed under these conditions (~5300 a.u. vs. ~2500 a.u. for control cells in the cytosol, and ~9100 a.u. vs. ~2400 a.u. in the nucleus) (Figure S6, Supplementary Materials). Similar to findings in cells infected under native conditions, >90% of active nuclear PIC showed strong CA signals when cells were exposed to Vpx.

## 4. Discussion

In this study, we provide a detailed analysis of early post-entry replication dynamics of HIV-1 in primary human macrophages, in order to identify steps that explain the slow and inefficient replication of the virus in this host cell type and to determine the effect of strategies frequently employed to enhance macrophage infection in experimental settings regarding the dynamics of events. The experiments were mostly performed with non-polarized (M0) macrophages, but similar infectivity and infection kinetics (not shown) were obtained for M2a macrophages, while M1-polarized macrophages were poorly infected with HIV-1.

Cytosolic entry of HIV-1 into differentiated macrophages, as assessed by inhibitor time-of-addition experiments, occurred more slowly than known for CD4^+^ lymphocytes. While productive HIV-1 entry into T-lymphocytes is generally completed within 2–4 h [6,7], resilience against the entry inhibitor MVC developed gradually with a half-time of roughly 8 h in MDMs in our experiments. A possible cause for this difference may be the route of entry used for productive infection. Whereas, in the case of CD4^+^ T cells, fusion at the plasma membrane [63] and possibly clathrin-mediated endocytosis [64] were reported as relevant entry routes, the process is less characterized in macrophages, and clathrin-independent uptake, e.g., macropinocytosis, may play a major role [65]. Dynamics and route of entry do not appear to be relevant for the slow replication in macrophages, however, since altering efficiency and route of cell entry by pseudotyping viral particles with the VSV-G glycoprotein did not notably affect the time course of subsequent replication steps, while enhancing overall infection rate. In contrast, the early post-entry events from reverse transcription of the viral RNA to integration represented a main bottleneck independent of the entry route, with reverse transcription and integration being completed only between two and three days p.i. as assessed by inhibitor time-of-addition experiments. Consistently, sensitive ddPCR detection of RT products revealed peak levels of early and late reverse transcripts around 72 h p.i. Interestingly, we observed no apparent lag phase between completion of reverse transcription and integration, suggesting that completion of viral DNA synthesis and/or entry of subviral complexes through the nuclear pore represent rate-limiting steps for early HIV-1 replication in macrophages.

These findings were corroborated and extended by quantitative microscopic analysis of productive subviral complexes in infected cells, using a previously established method [50]. Consistent with the low efficiency of HIV-1 infection generally observed in macrophages [11,66], the majority of IN.eGFP-positive objects were detected in the cytosol and only a small minority were associated with detectable products of reverse transcription. Furthermore, 90% or more of all intracellular IN.eGFP-positive complexes were found to be cytoplasmic at all times, and only 5% of all intracellular IN.eGFP-positive structures became EdU-positive during the course of three days. Thus, abortive HIV-1 infection of MDMs mainly stalls prior to efficient reverse transcription, and replication may be limited at the stage of cytosolic entry (despite using a highly efficient R5-tropic Env from a primary brain-derived HIV-1 isolate [36]) and/or reverse transcription. In accordance with the findings from our other experiments, the number of EdU-positive objects increased over the first two days of observation and a gradual shift of objects into the nucleus occurred within that time frame. As observed earlier by us [50], most of the nuclear complexes in macrophages were associated with a strong signal for the viral CA protein.

At the earliest detection time of 24 h p.i., a relatively high proportion of subviral complexes were already observed close to the nuclear envelope. Although the resolution of confocal microscopy does not allow to state with certainty that these complexes were directly associated with the nuclear envelope, the data support the hypothesis that passage of viral complexes through the nuclear pore presents a kinetic bottleneck, while trafficking toward the nuclear envelope appears to be rapid. Our microscopic approach also allowed us to correlate the subcellular localization of complexes with the EdU click-labeling signal intensity as a relative measure of reverse transcription. Overall, a strong peak population of EdU-positive complexes displayed low EdU click-labeling intensities, complemented by a broadly distributed population of higher intensity complexes. Although the intensity levels cannot be directly correlated to the length of the respective RT products, this finding points to a lag phase between early and late reverse transcription, resulting in relative accumulation of complexes stalled at an early state of the process. Productive RTC/PIC were detected at all subcellular localizations analyzed; however, while they represented a small minority in the cytosolic area—consistent with a high number of non-productive uptake events under these conditions—their proportion in the nucleus was much higher at all time points tested. Although blocking reverse transcription does not prevent import of HIV-1 subviral complexes into the nucleus [54,55], this observation may hint at a dynamic correlation between reverse transcription and nuclear import. The proportion of EdU-positive complexes found in the nucleus increased from 11% at 24 h to >50% of all productive RTC/PIC at later time points, while only ~3% of all IN.eGFP-positive complexes reached the nucleus during this time period.

Nuclear PIC also displayed higher overall EdU click-labeling intensities than EdU-positive complexes detected in the cytosolic area, suggesting that nuclear import kinetics is either correlated to the progression of reverse transcription, or that reverse transcription may be completed within the nucleus in this cell type. Interestingly, while a population of EdU click-labeling high-intensity objects was present in the cytoplasm, as well as in the nucleus, complexes detected close to the nuclear envelope almost exclusively displayed low-intensity EdU click-labeling signals. While this observation needs to be interpreted with some caution due to the low number of complexes detected at that site in our analyses (*n* = 92 in 172 cells from four donors), it would be in agreement with the hypothesis that a delay or block of reverse transcription impairs nuclear import kinetics [67,68]. Alternatively, this population may represent dead-end complexes with functionally unrelated or related defects in reverse transcription and nuclear import. The inference of reverse transcription and nuclear import dynamics from the microscopic data is currently limited by the fact that different samples fixed at different time points are compared in these bulk experiments. A definitive conclusion concerning the relationship between reverse transcription and nuclear import awaits the development of live-cell compatible detection approaches suitable for single-particle tracking.

The stimulatory effect of Vpx-mediated SAMHD1 degradation on HIV-1 infection efficiency in macrophages—presumably through increasing intracellular dNTP levels—is well documented [23,24,25,29,69]. Our dynamic analyses and quantitative microscopy clearly show that not only efficiency of reverse transcription (i.e., the number of productive complexes and the amount of RT products) was enhanced, but also the kinetics of reverse transcription were much more rapid in cells pre-exposed to Vpx_mac239_. These results support the conclusion that removal of this kinetic obstacle—most likely by increasing dNTP levels in MDMs—is the underlying cause of the enhanced infectivity observed under these conditions. This effect was much less obvious when deoxynucleosides were added prior to infection, which only yielded increased RT products at 36 h p.i. and later. This delayed effect may be due to the need for uptake and conversion into the dNTP, but could also reflect a threshold dNTP level for rapid and efficient reverse transcription, which may be difficult to achieve in the presence of SAMHD1.

Even upon SAMHD1 degradation, however, copy numbers of early and late RT products were still low at 4–8 h in macrophages, not matching the fast time course observed for T cells [17,70]. We cannot currently discriminate whether this simply reflects the increased contribution of the slow entry phase on the overall dynamics or points to a remaining post-entry lag phase under these conditions; mathematical modeling of data with the aim of disentangling these aspects is currently under way. A rapid loss of inhibitor sensitivity is seen for both EFV and RAL after 8 h with almost complete loss of inhibition at 24 h in the presence of Vpx_mac239_, compared to 2–3 days required for completion of reverse transcription and integration without pre-exposure to Vpx. Loss of inhibitor sensitivity occurred largely in parallel for RT and IN inhibitors independent of Vpx addition, indicating that, even under conditions where the rate of reverse transcription is significantly enhanced, reverse transcription and/or nuclear entry remain rate-limiting. Time-resolved analysis of both processes will be needed to resolve the actual block, but our combined data indicate that completion of reverse transcription and nuclear entry may be more closely linked, and synthesis of HIV-1 cDNA may largely be completed at the nuclear pore or inside the nucleus in primary human macrophages.

## Figures and Tables

**Table 1:** List of sequences and source of primers and probes used to detect human immunodeficiency virus type 1 (HIV-1) reverse transcriptase (RT) products with digital droplet PCR (ddPCR). LTR—long terminal repeat; Fw—forward; Rv—reverse; FAM—fluorescein amidite; BHQ1—black hole quencher 1.

Target	Sequence (5′–3′)	Reference
5′-LTR Fw	TTAAGCCTCAATAAAGCTTGCC	[43]
5′-LTR Rv	GTTCGGGCGCCACTGCTAG	
5′-LTR Probe	FAM–CCAGAGTCACACAACAGACGGGCA–BHQ1	
*Gag* Fw	CATGTTTTCAGCATTATCAGAAGGA	[44]
*Gag* Rv	TGCTTGATGTCCCCCCACT	
*Gag* Probe	HEX—CCACCCCACAAGATTTAAACACCATGCTAA–BHQ1	
2-LTR Fw	CTAACTAGGGAACCCACTGCT	[45]
2-LTR Rv	GTAGTTCTGCCAATCAGGGAA	
2-LTR Probe	FAM—AGCCTCAATAAAGCTTGCCTTGAGTGC–BHQ1	
*RPP30* Fw	GATTTGGACCTGCGAGCG	[42]
*RPP30* Rv	GCGGCTGTCTCCACAAGT	
*RPP30* Probe	FAM–CTGACCTGAAGGCTCT–BHQ1	

## Supplementary Materials

The following are available online at http://www.mdpi.com/1999-4915/10/11/620/s1. Figure S1: Macrophage polarization and kinetics of early HIV-1 replication in cells from individual donors; Figure S2: Infectivity values in cells from individual donors summarized in Figure 2 and absolute copy numbers of RT products; Figure S3: Distribution of mean EdU signal intensities of individual HIV-1 RTC/PIC; Figure S4: SAMHD1 degradation induced by transduction with VLPs carrying Vpx_mac239_; Figure S5: Quantitation of RT products in cells transduced with vectors lacking Vpx; Figure S6: Mean EdU signal intensities of HIV-1 RTC/PIC after addition of Vpx_mac239_.
