# Supplementary material for: Detailed Characterization of Early HIV-1 Replication Dynamics in Primary Human Macrophages
Source: Viruses. 2018 Nov 10;10(11):620. doi: 10.3390/v10110620 (PMC6266216; doi:10.3390/v10110620)
Supplement: Supplementary file 1 [file viruses-10-00620-s001.pdf]

Supplementary Materials

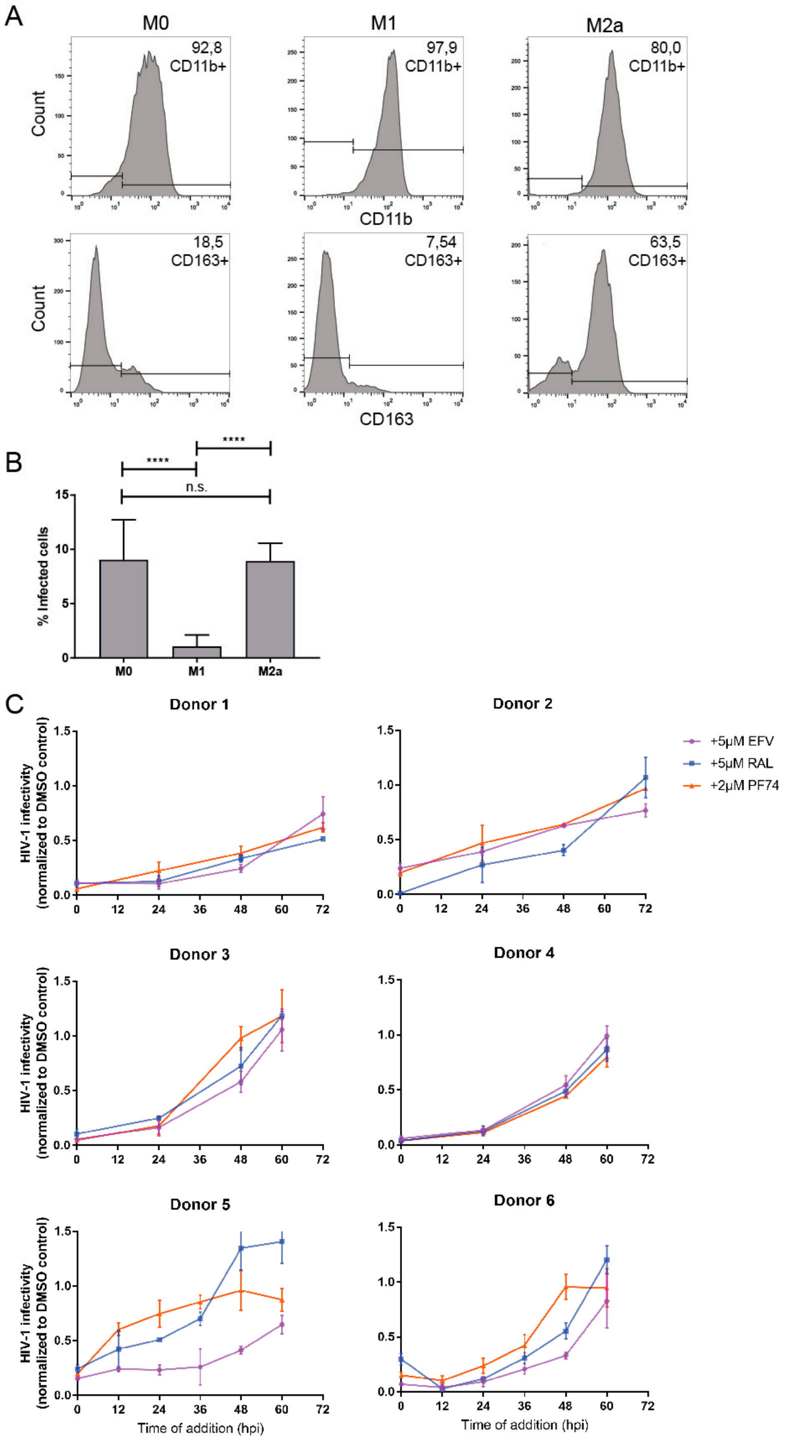

Figure S1. Related to Figure 1. Macrophage polarization and kinetics of early HIV-1 replication in cells from individual donors. (A) Monocytes were differentiated and polarized with human AB serum (M0), GM-CSF and IFNγ (M1) or M-CSF and IL-4 (M2a). Cells were fixed and stained with fluorescently labeled antibodies against the indicated

markers and analyzed by flow cytometry. **(B)** Monocytes from two donors were isolated from healthy PBMC and differentiated into polarized macrophages using either human AB serum (M0), GM-CSF and IFNɣ (M1) or M-CSF and IL-4 (M2a). Subsequently cells were infected with R5-pseudotyped HIV-1NL4-3 in triplicates. At 24h p.i., medium was changed and 5µM MVC was added to the medium. At day 6 p.i. cells were fixed, and proportions of infected cells were scored. The graphs show proportions of infected cells. Error bars represent SD of the fraction of infected cells of the triplicates of both donors. Statistical significance was assessed by two-tailed non-paired Mann-Whitney test or non-paired student´s t test; ****: p<0.0001, n.s.: not significant. **(C)** MDM were infected with R5-pseudotyped HIV-1NL4-3 as in Figure 1. 5µM EFV (purple), 5 µM RAL (blue) or 2µM PF74 (orange) was added at the indicated time points. Medium was changed, and infection continued until day 6 p.i. Cells were fixed, and infectivity was scored by immunostaining with anti-CA antiserum and counterstaining with Hoechst.

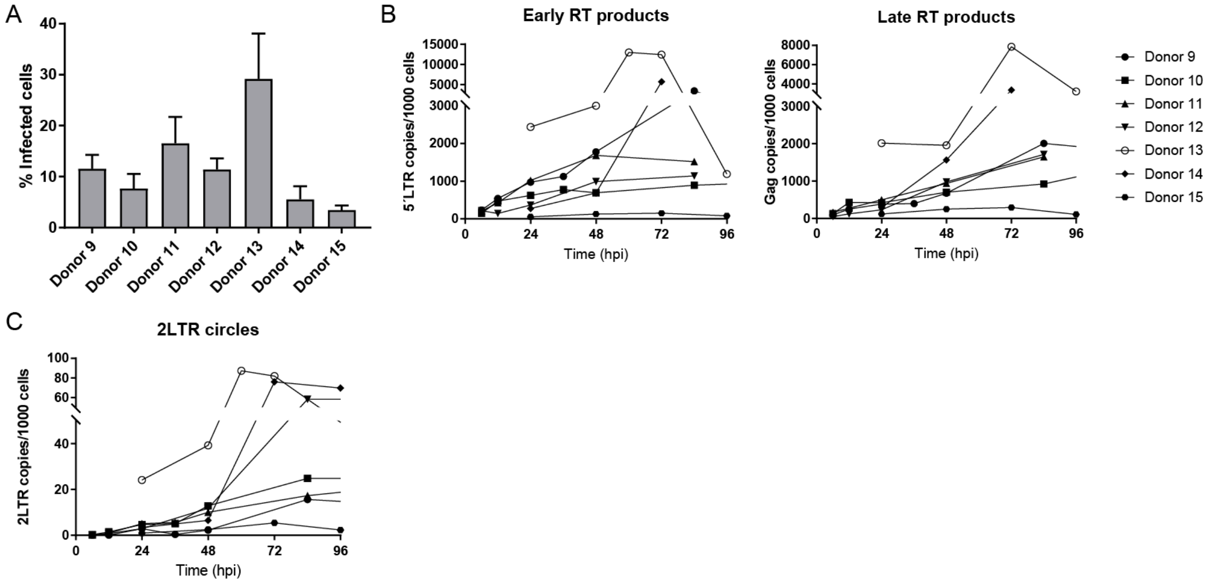

Figure S2. Related to Figure 2. Infectivity values in cells from individual donors summarized in Figure 2 and absolute copy numbers of RT products. MDM from seven donors were infected with R5-pseudotyped HIV-1NL4-3 as explained in Fig. 2. After 24h, medium was changed and 5µM MVC was added. **(A)** Medium was changed at 3d p.i. and infectivity was scored at day 6 after infection. The graph shows the percentage of infected cells from individual donors summarized in **Fig. 2** (mean values and SD from triplicate infections). **(B, C)** Infected cells were harvested at the indicated time points and HIV-1 RT products were quantitated in cell lysates by ddPCR as detailed in Materials and Methods. Copy numbers of early and late RT products (B) or 2-LTR circles (C) were normalized to the copy numbers of the housekeeping gene at different time points p.i. from each donor. Each symbol represents a donor.

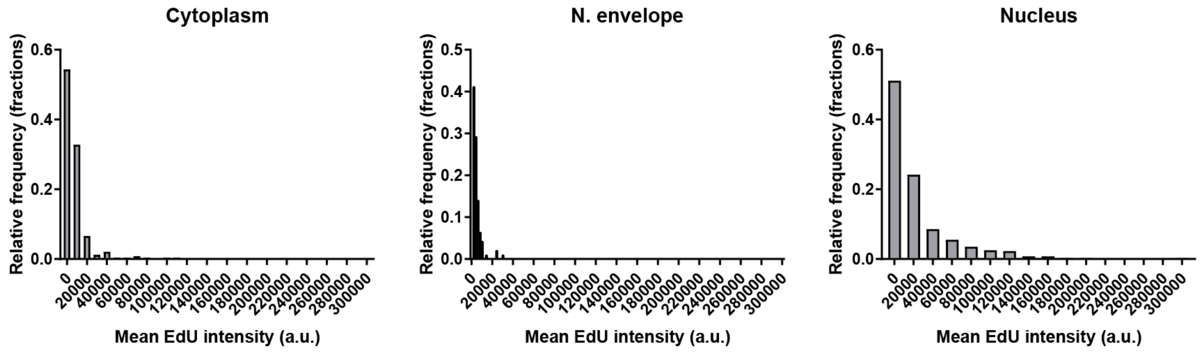

Figure S3. Related to Figure 3. Distribution of mean EdU signal intensities of individual HIV-1 RTC/PIC. MDM from four donors were infected with HIV-1NL4-3 (IN.eGFP) in the presence of 10µM EdU. Cells were fixed at 48h p.i., click-labeled and immunostained with anti-CA and anti-laminA/C antibodies. Histogram shows frequency distribution of mean EdU signal intensities of individual RTC/PIC at three subcellular localizations. Black lines represent the mean. Statistical significance was assessed by two-tailed non-paired Mann-Whitney test; Cytoplasm vs. Nucleus: ****; Cytoplasm vs. N.envelope: *; N.envelope vs. Nucleus: ****; *:p=0.02, ****:p<0.0001.

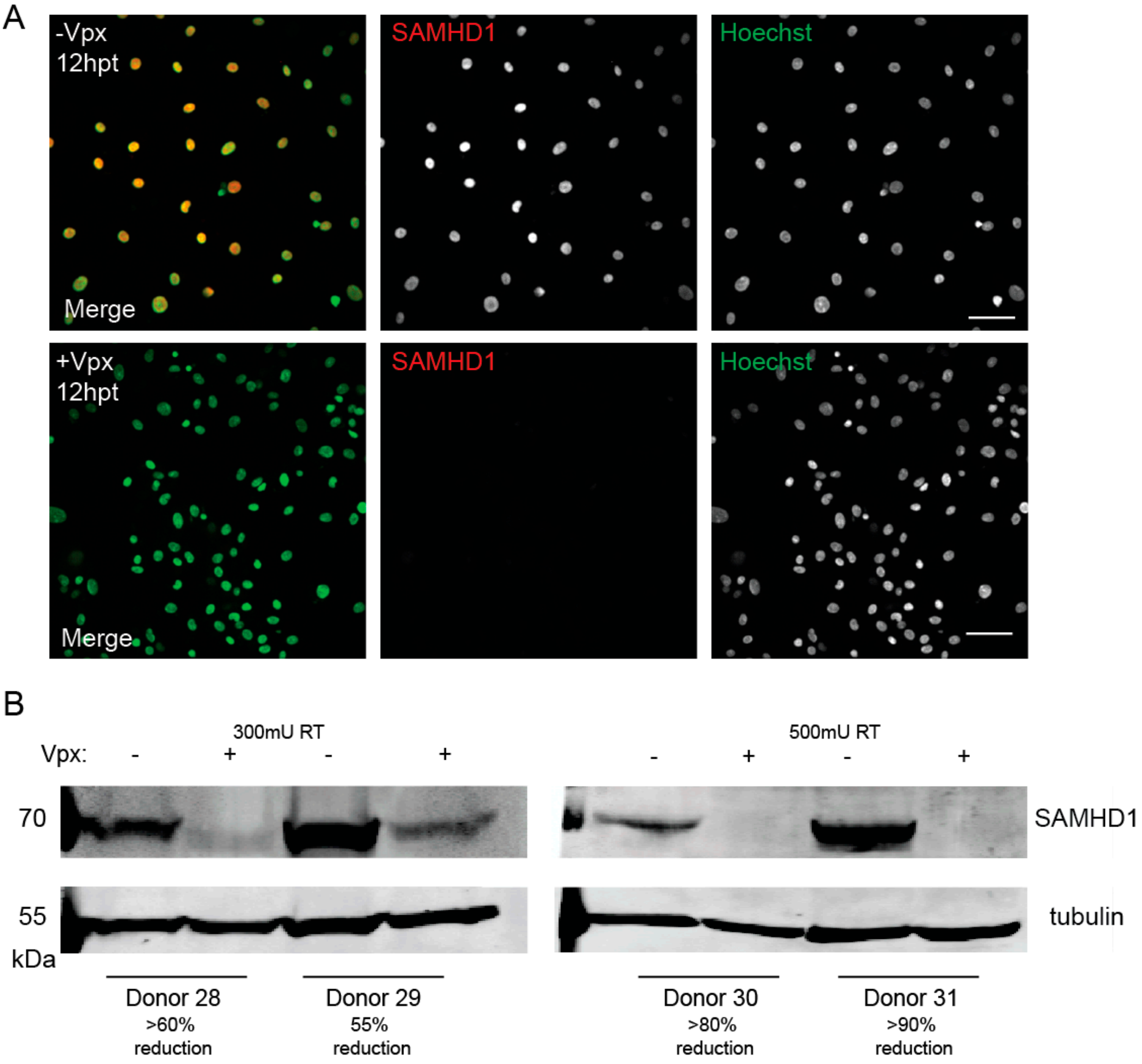

Figure S4. Related to Figures 5-7. SAMHD1 degradation induced by transduction with VLPs carrying Vpx$_{mac239}$. (A) Single-cell analysis. For experiments involving single cell analysis, MDM of four donors were transduced with 150mU RT of either VLPs lacking (upper panel) or carrying (lower panel) Vpx$_{mac239}$. At 12h p.t. cells were fixed and immunostained with antibody against SAMHD1 (red) and counterstained with Hoechst (green). Scale bars represent 30µm. **(B)** MDM of four donors were transduced with 300 or 500mU RT of VLPs lacking or carrying Vpx$_{mac239}$. Cell lysates harvested after 12h were analyzed for SAMHD1 expression by quantitative immunoblot (Licor) using antibodies against SAMHD1 as well as against α-tubulin as a loading control. Positions of molecular mass standards

are shown to the left. Relative reduction of SAMHD1 bulk levels was quantitated from the band intensities determined using LiCor Odyssey software.

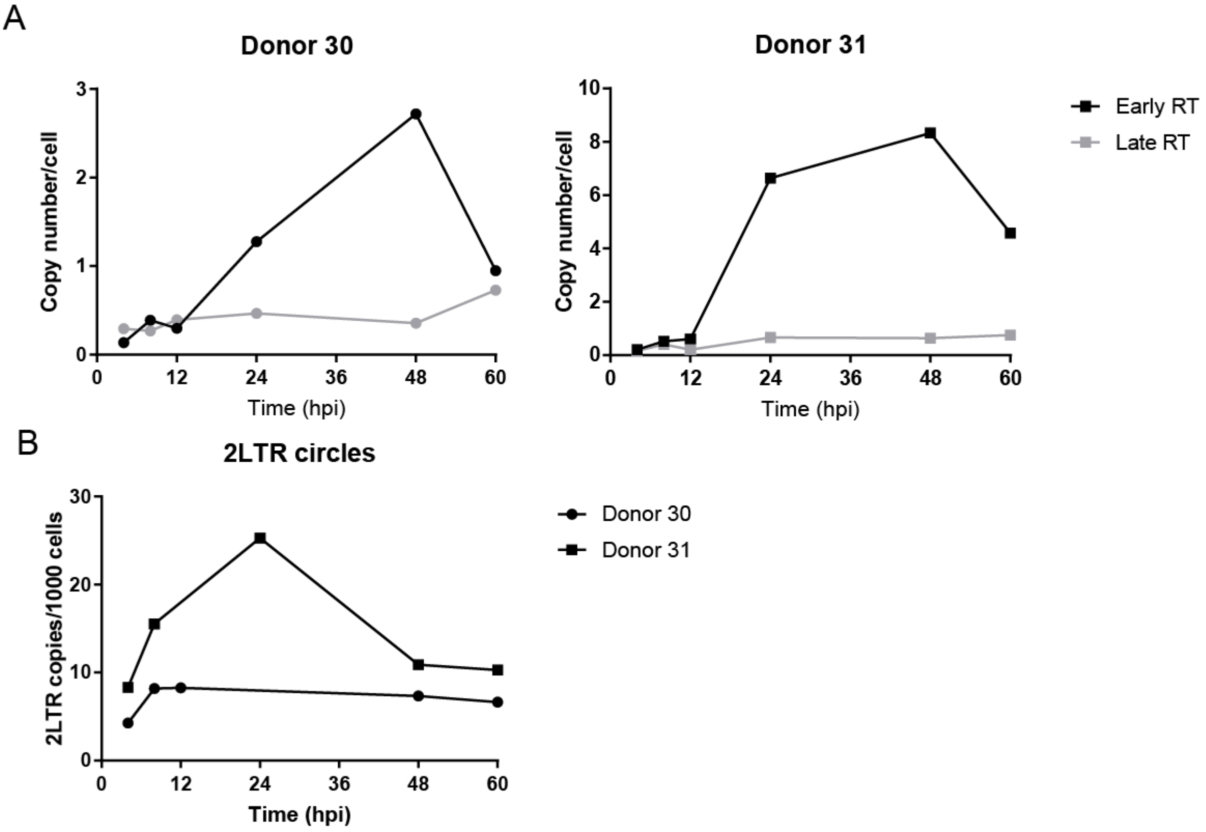

Figure S5. Related to Figure 6. Quantitation of RT products in cells transduced with vectors lacking Vpx. MDM from two donors were transduced with VLPs lacking Vpx. After 12h, cells were infected with 50ng p24 R5-pseudotyped HIV-1_{NL4-3}. At 24h p.i., medium was replaced and 5µM MVC was added. Cells were lysed at the indicated time points and RT products were detected by ddPCR as described in Materials and Methods. Absolute copy numbers of early (black lines), late RT (gray lines) products **(A)** and 2-LTR circles **(B)** at each time point p.i. were normalized to the copy numbers of the housekeeping gene. Each symbol shape represents a donor. Notice that detection of 2-LTR circles was not efficient, possibly due to the low infectivity observed in the absence of Vpx.

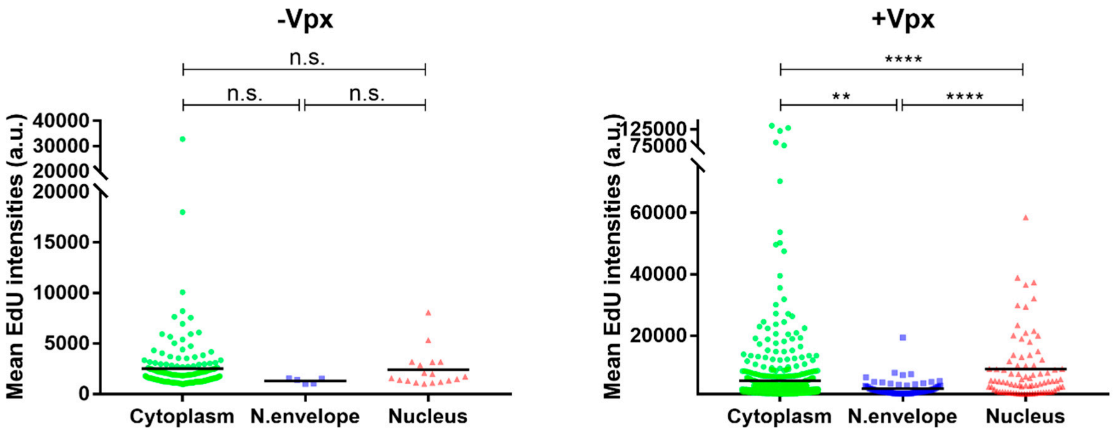

Figure S6. Related to Figure 7. Mean EdU signal intensities of HIV-1 RTC/PIC after addition of Vpx$_{mac239}$. MDM from four donors were transduced with VLP lacking or carrying Vpx$_{mac239}$. After 12h, medium was changed and cells were infected with R5-pseudotyped HIV-1$_{NL4-3}$ (IN.eGFP) in the presence of 10µM EdU. Cells were fixed at 24h p.i., click-labeled and immunostained with anti-CA and anti-laminA/C antibodies. Plot shows mean EdU signal intensities of individual RTC/PIC at three subcellular localizations from the 24h data set of Fig. 7. Black lines represent the mean. Statistical significance was assessed by two-tailed non-paired Mann-Whitney test; **:p=0.003, ****:p<0.0001, n.s.: not significant.
